# Identification of alternative splicing events related to fatty liver formation in duck using full-length transcripts

**DOI:** 10.1186/s12864-023-09160-4

**Published:** 2023-03-01

**Authors:** Yiming Wang, Linfei Song, Mengfei Ning, Jiaxiang Hu, Han Cai, Weitao Song, Daoqing Gong, Long Liu, Jacqueline Smith, Huifang Li, Yinhua Huang

**Affiliations:** 1 State Key Laboratory for Agrobiotechnology, College of Biology Sciences, China Agricultural University, No.2 Yuan Ming Yuan West Road, Hai Dian District, Beijing, 100193 China; 2 Department of Waterfowl Breeding and Production, Jiangsu Institute of Poultry Science, No. 58 Cangjie Road, Hanjiang District, Yangzhou, 349019093 China; 3 College of Animal Science and Technology, Yangzhou University, Yangzhou, China; 4 The Roslin Institute and Royal (Dick) School of Veterinary Studies, University of Edinburgh, Midlothian, EH25 9RG UK

**Keywords:** Full-length transcripts sequencing, Duck, Fatty liver, Alternative splicing

## Abstract

**Background:**

Non-alcoholic fatty liver disease (NAFLD) is one of most common diseases in the world. Recently, alternative splicing (AS) has been reported to play a key role in NAFLD processes in mammals. Ducks can quickly form fatty liver similar to human NAFLD after overfeeding and restore to normal liver in a short time, suggesting that ducks are an excellent model to unravel molecular mechanisms of lipid metabolism for NAFLD. However, how alternative splicing events (ASEs) affect the fatty liver process in ducks is still unclear.

**Results:**

Here we identify 126,277 unique transcripts in liver tissue from an overfed duck (77,237 total transcripts) and its sibling control (69,618 total transcripts). We combined these full-length transcripts with Illumina RNA-seq data from five pairs of overfed ducks and control individuals. Full-length transcript sequencing provided us with structural information of transcripts and Illumina RNA-seq data reveals the expressional profile of each transcript. We found, among these unique transcripts, 30,618 were lncRNAs and 1,744 transcripts including 155 lncRNAs and 1,589 coding transcripts showed significantly differential expression in liver tissues between overfed ducks and control individuals. We also detected 27,317 ASEs and 142 of them showed significant relative abundance changes in ducks under different feeding conditions. Full-length transcript profiles together with Illumina RNA-seq data demonstrated that 10 genes involving in lipid metabolism had ASEs with significantly differential abundance in normally fed (control) and overfed ducks. Among these genes, protein products of five genes (*CYP4F22*, *BTN*, *GSTA2*, *ADH5*, and *DHRS2* genes) were changed by ASEs.

**Conclusions:**

This study presents an example of how to identify ASEs related to important biological processes, such as fatty liver formation, using full-length transcripts alongside Illumina RNA-seq data. Based on these data, we screened out ASEs of lipid-metabolism related genes which might respond to overfeeding. Our future ability to explore the function of genes showing AS differences between overfed ducks and their sibling controls, using genetic manipulations and co-evolutionary studies, will certainly extend our knowledge of genes related to the non-pathogenic fatty liver process.

**Supplementary Information:**

The online version contains supplementary material available at 10.1186/s12864-023-09160-4.

## Background

In humans, non-alcoholic fatty liver disease (NAFLD) is one of the most common global diseases with the overall incidence being 46.9 cases per 1000 people per year in a recent survey [1]. NAFLD has clinical-pathological symptoms including isolated steatosis, non-alcoholic steatohepatitis (NASH) and liver fibrosis [2]. The possibility of NAFLD is higher in men and increases with advancing age. NAFLD in obese children and adolescents further develops into more serious diseases [3, 4]. Similarly, intake of energy-rich food induces fatty liver in ducks, which shows the same pathology with human NAFLD [5, 6]. However, ducks can recover from fatty liver quickly and protect their liver against pathological changes such as fibrosis and ultimately cirrhosis that frequently happen in human NAFLD [7–9]. Therefore, ducks provide a good model to unravel the molecular mechanisms underlying lipid metabolism and hepatic steatosis.

Alternative splicing is one of the most important events that regulate function of proteins through generating different transcript isoforms. Alternative splicing has been reported in many bioprocesses including human ageing, human cancer and sex selection of birds [10–12]. Alternative splicing also plays an important role in NAFLD and dysregulation of alternative splicing contributes to development of NAFLD [13–15]. For example, DRAK2 inhibits SRPK1-mediated SRSF6 phosphorylation and leads to changes of SRSF6-associated alternative splicing of mitochondrial function-related genes to aggravate NALFD procedure [16]. To our knowledge, studies on fatty liver of ducks have been focused on genes related to lipid metabolism based on RNA-seq short read data or explored nutrition complement affecting fat deposition in duck liver. [17–20]. However, the role of alternative splicing is unclear in process of responding to fatty liver of duck. Here we performed full-length transcript sequence using a pair of sibling ducks, which were fed with high fat corn feed and commercial forage, respectively. We annotated transcripts and compared their expression in liver tissues of overfed and control ducks. This effort identified 27,317 ASEs, with 142 of them having significant frequency changes in liver tissues between overfed and control ducks. Moreover, we have identified five lipid metabolism related genes (*CYP4F22*, *BTN*, *GSTA2*, *ADH5*, and *DHRS2* genes) from these 142 events. These observations revealed the probable ASEs regulating the formation and defense process of liver in avoiding pathogenic fatty liver disease.

## Results

### PacBio full-length high-coverage liver transcriptomic profile of overfed and control ducks

**Fig. 1:**
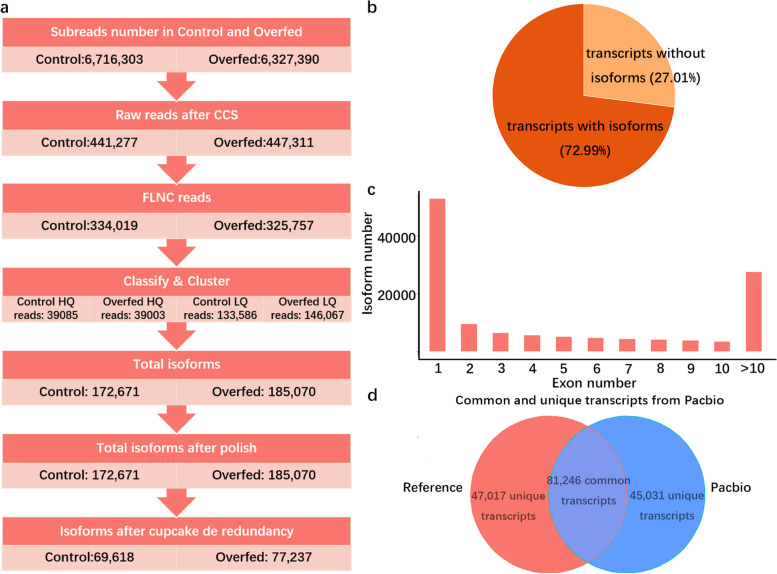
Transcript processing workflow and statistics. **a** Procedure of total transcripts access for ducks. **b** The ratio of transcripts with multiple isoforms and unique transcripts without other isoforms. **c** The number of transcript isoforms with different exon number. **d** Venn diagram showing common and unique transcripts with or without reference genes

We sequenced liver transcriptomes of a sib-pair ducks using the PacBio Sequel platform. This identified 172,671 and 185,070 full-length transcripts from 6,327,390 subreads of overfed duck and 6,716,303 subreads of control individuals, respectively. We then identified 77,237 and 69,618 unique transcripts from liver tissues of overfed and control ducks respectively, and merged these transcripts into a single data set containing 126,277 unique transcripts (Fig. 1a). Alignment of 77,237 and 69,618 liver transcripts of overfed and control ducks to our duck reference gene set showed that 9,554 and 9,515 genes were expressed, respectively. These numbers of expressed genes covered 82.05% and 82.88% genes detected by Illumina RNA-seq data in overfed and control ducks (unpublished data), suggesting that these two full-length transcriptomes were high coverage and provided a reasonable substrate for the analysis presented in this study. Sequence alignment of 126,277 unique transcripts to our new duck assembly (SKLA1.0, PRJNA792297) and gene reference set showed that 27.01% of them were unique transcripts, while 72.99% have different transcripts (Fig. 1b). The average number of exons was 6.4, the average length of these 126,277 unique transcripts 3,864 bp and 27,410 transcripts had more than 10 exons (Fig. 1c). When compared to our duck reference gene sets, a total of 81,246 transcripts were mapped to 10,888 genes and 45,031 transcripts were novel transcripts (Fig. 1d). Among these 45,031 novel transcripts, 30,512 were annotated as novel transcripts of lncRNAs and 14,519 were annotated as novel transcripts of coding genes. Moreover, 302 transcripts were intra-chromosomal fusion transcripts. These data suggested that our full-length transcriptome was a rich source of biological diversity.

### Comparison of transcript expression between overfed and control ducks

**Fig. 2:**
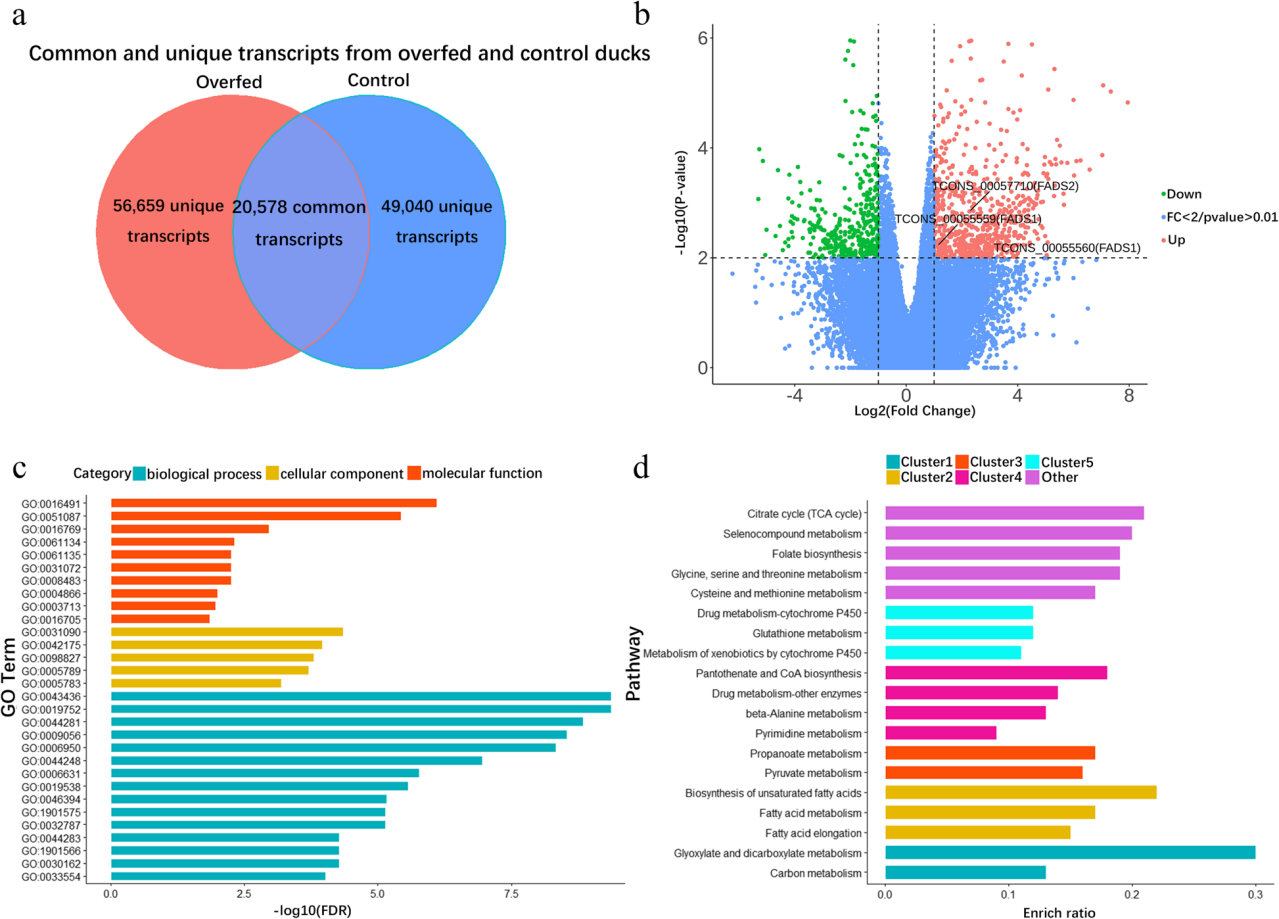
Analysis of differentially expressed transcripts. **a** Venn diagram of unique and common transcripts of overfed and control groups. **b** Volcano plot for differentially expressed transcripts (FC > 2, *p*-value < 0.01 in up class, FC < 0.5, *p*-value < 0.01 in down class). **c** GO enrichment analysis of genes with significantly differentially expressed transcripts. **d** KEGG enrichment analysis of genes with significantly differentially expressed transcripts

We compared full-length liver transcriptomic profiles of the above sib-pair ducks. This effort found 56,659 transcripts uniquely presented in overfed ducks, 49,040 transcripts only presented in control ducks and 20,578 transcripts were observed in both overfed and control ducks (Fig. 2a). We further counted expression levels of transcript by TPM (Transcripts per kilobase of exon model divided by million mapped reads) and identified 1,744 transcripts of 1,282 genes showing significantly differential expression (DETs, *p*-value < 0.01) in liver tissues between overfed and control ducks (Additional file 1: Table S1). Among 1,744 DETs, 982 were upregulated and 762 were downregulated with *p*-value < 0.01 in overfed ducks when compared to those in control ducks (Fig. 2b). Using thresholds of |log2FC|> 1 (FC, Fold Change), we identified 683 being upregulated and 382 being downregulated in overfed ducks when compared to their sibling controls. Gene ontology (GO) analysis indicated that 1,282 genes presenting DETs were enriched in 45 biological functions, with 27 involved in fatty acid metabolic process (GO:0,006,631) with FDR < 0.05 (Fig. 2c). KEGG analysis demonstrated that 9 genes showing DETs were enriched in biosynthesis of unsaturated fatty acids, fatty acid metabolism and fatty acid elongation pathway (*p*-value < 0.01, Fig. 2d). *FADS1* (Fatty Acid Desaturase 1) and *FADS2* (Fatty Acid Desaturase 2) were previously reported to reduce lipid accumulation and influence the NAFLD process in mice [21–25]. Interestingly, we found that transcript isoforms of *FADS1* (Fatty Acid Desaturase 1) TCONS_00055559 and *FADS2* (Fatty Acid Desaturase 2) TCONS_00057710 were significantly upregulated in overfed ducks when compared to those in control ducks. These results reveal detailed information of expression profiles of *FADS1* and *FADS2* at the transcript-level and identify the main transcripts of *FADS1* and *FADS2* which might function in the formation of fatty liver in ducks to alleviate liver injury. Moreover, we compared reference transcripts to the above 1,744 differentially expressed transcripts to verify the confidence of detected transcripts. We found 893 of these DETs, including TCONS_00057710, were known transcripts of *FADS1*, while TCONS_00055559 was a novel transcript of *FADS1*. Aligning all four reference FADS1 protein sequences to TCONS_00055559 protein sequence, we found that TCONS_00055559 was a new recombination of FADS1 exons. This observation suggested that TCONS_00055559 was a new transcript of FADS1 in ducks (Additional file 2: Fig S1 and Additional file 3: Table S2).

### Prediction of lncRNA and lncRNA-coding cis-acting pairs

**Fig. 3:**
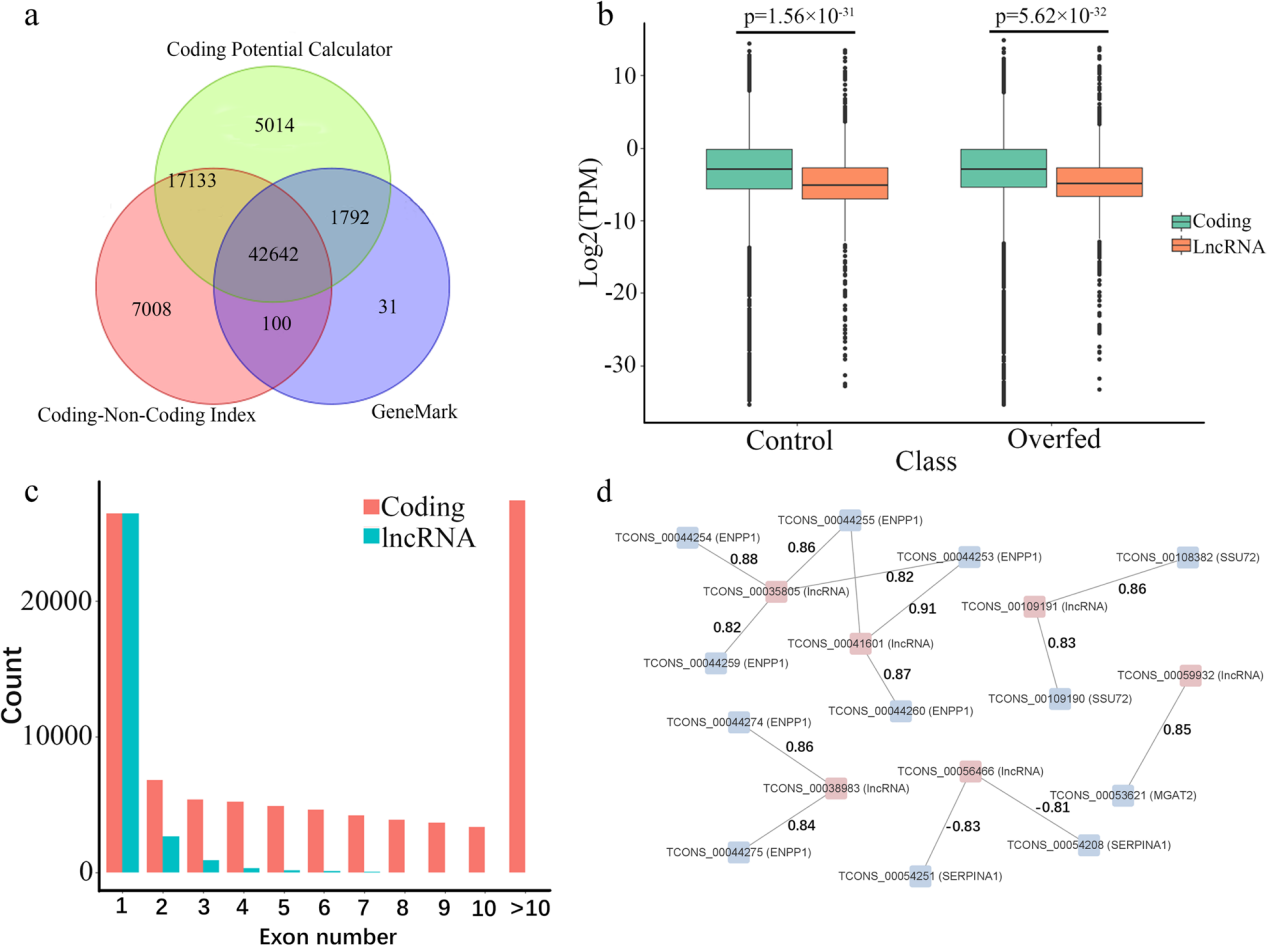
Identification and characteristics of lncRNA. **a** Venn diagram of non-coding transcripts predicted by GeneMark, CPC, and CNCI. **b** Expression level of transcripts of coding genes and lncRNA in overfed and control groups. **c** The number of lncRNA with different exon number **d** Correlated cis-acting pairs of DETs from lncRNA and neighbouring coding genes within 10 kb

For the above 126,277 unique transcripts, 42,642 transcripts were predicted as non-coding sequences and 30,618 were annotated as lncRNA, including 155 DETs (Fig. 3a). We compared characteristics of lncRNA and protein-coding transcript isoforms. We found that lncRNA had lower mean expression level (TPM) than protein-coding transcripts did, in both control and overfed ducks (Fig. 3b). Among 30,618 lncRNA transcripts, 12,861 did not overlap with coding genes and 17,757 did overlap with coding-genes. Detailed transcript structure analysis indicated, among these lncRNA transcripts, a few (2.13%) had more than three exons, a small percentage (11.64%) had two or three exons, and many (86.23%) had only one exon. This was different from the case of protein-coding transcripts, where most (59.70%) had more than three exons, a few (12.68%) had two or three exons and the remainder (27.62%) had only one exon (Fig. 3c). Moreover, we calculated the correlation between 155 lncRNA DET and adjacent protein-coding transcripts with 10 liver tissue RNA-seq transcriptomes. This analysis identified 57 lncRNA-coding cis-acting pairs, including 34 lncRNA and 52 protein-coding transcripts from 32 genes with a Pearson correlation higher than 0.8. Amongst these pairs, four genes (*ENPP1* (ectonucleotide pyrophosphatase 1), *SERPINA1* (serpin family A member 1), *MGAT2* (alpha-1,6-mannosyl-glycoprotein 2-beta-N-acetylglucosaminyltransferase) and *SSU72* (RNA polymerase II CTD phosphatase) were reported to have close association with NAFLD process (Fig. 3d). Overexpression of *ENPP1* in mice leads to insulin resistance and *MGAT2* deficiency reduces lipid absorption and insulin resistance [26, 27]. *SERPINA1* was associated with severity of NAFLD and *SSU72* influenced NAFLD deterioration [28, 29]. It will therefore be of interest to study whether and how lncRNA interacts with these four genes to regulate the fatty liver process of ducks.

### Identification of ASEs using duck full-length transcripts

Alternative splicing always requires the spliceosome, which catalyzes splicing reactions [30]. Under the function of the spliceosome, transcripts undergo one or more forms of alternative splicing. We counted ASEs which included skipped exon (SE), mutually exclusive exons (MX), alternative 5’splice site (A5), alternative 3’splice site (A3) and retained intron (RI) (Additional file 2: Fig S2a). Among the above 126,277 unique transcripts, we detected 27,317 ASEs in 5,665 genes, which included 18% RI, 41% SE, 19% A3, 20% A5 and 2% MX (Additional file 2: Fig S2b). We aligned transcripts to our duck reference genome to define ASEs as known and novel classes. This found 26,979 ASEs in known reference genes and 338 ASEs in novel genes.

**Fig. 4:**
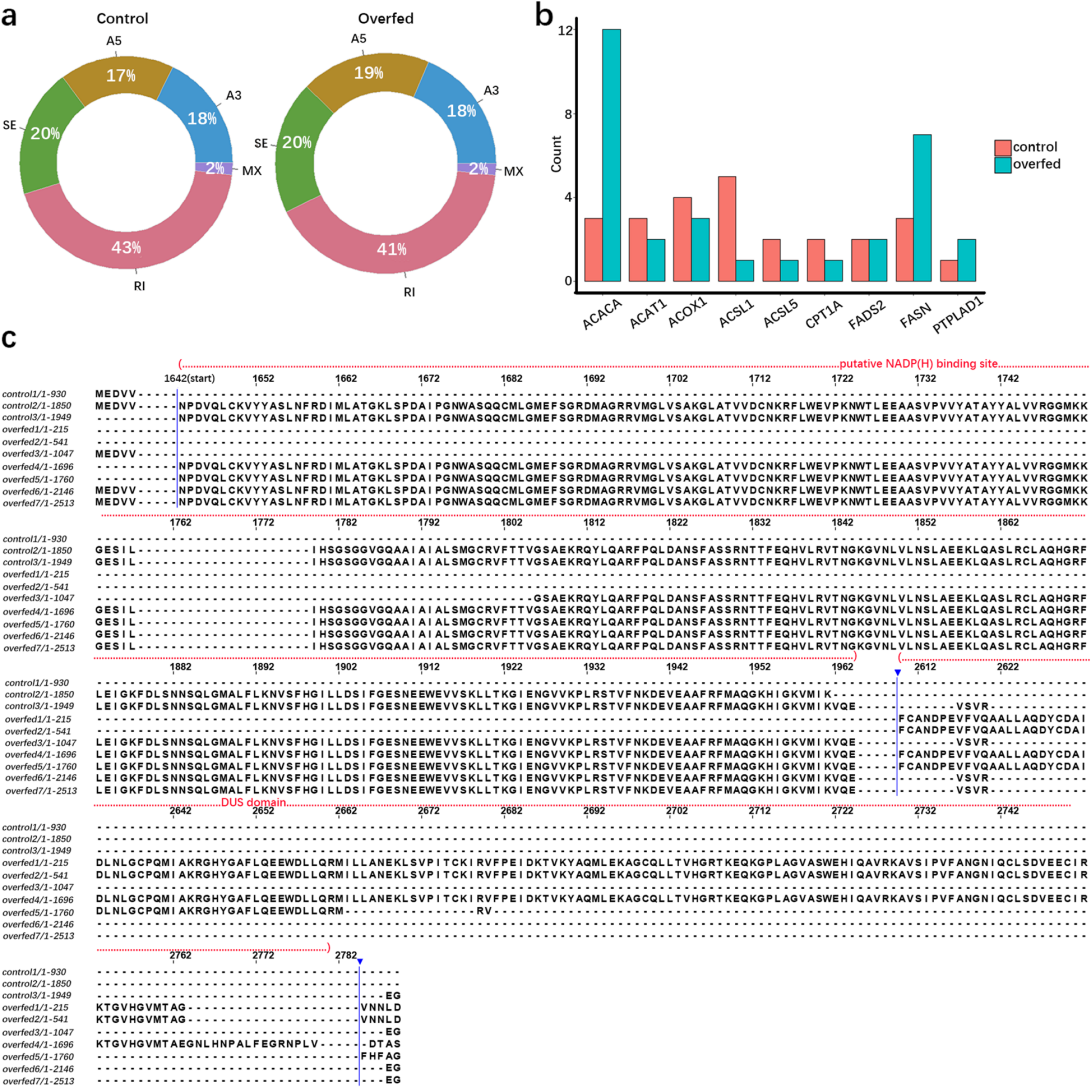
Statistics of ASEs and analysis of ASEs in fatty acid metabolism genes. **a** The ratio of each ASEs class in overfed and control groups. **b** Transcript numbers of nine fatty acid related genes in overfed and control ducks. **c** Protein sequence alignment of FASN transcripts from overfed and control ducks

We then compared ASE characteristics in liver transcriptomes of overfed and control ducks. This analysis detected 20,823 ASEs in liver of control and 26,228 in overfed ducks. Amongst these, the liver of control duck had a large proportion of RI events (43%), a small proportion of SE (20%), A3 (18%) and A5 (17%), and a few MX (2%). This is similar to the case in overfed ducks, where there was 41% RI, 20% SE, 18% A3, 19% A5 and 2% MX events (Fig. 4a). We then compared the number of transcript isoforms in liver transcriptomes and found that 9 genes (*ACAT1* (acetyl-CoA acetyltransferase 1), *ACSL1* (acyl-CoA synthetase long chain family member 1), *CPT1A* (carnitine palmitoyltransferase 1A), *FADS2* (fatty acid desaturase 2), *ACSL5* (acyl-CoA synthetase long chain family member 5), *PTPLAD1* (3-hydroxyacyl-CoA dehydratase 3), *FASN* (fatty acid synthase), *ACOX1* (acyl-CoA oxidase 1) and *ACACA* (acetyl-CoA carboxylase alpha)) had different numbers of protein sequences between overfed and control ducks (Fig. 4b). Among them, *FASN* was a key enzyme in fatty acid biosynthesis, bound to FMN (Flavine Mononucleotide) cofactor via its DUS (Dihydrouridine synthase) domain to produce reactive oxygen species (ROS) in NADPH-dependent oxidation [31]. Interestingly, we found that *FASN* had a transcript (‘overfed4’ in Fig. 4c and Additional File 3: Table S3) which contained the DUS domain and expressed in livers of five overfed ducks, but was not detected in these of five control ducks (Fig. 4c). These observations together with overfed ducks with fatty liver did not present inflammation and fibrosis (unpublished data) suggesting that ducks might relieve the oxidative damage of fatty liver through AS of these genes.

### Significantly changed ASEs involved in lipid metabolism

Transcript isoforms can have similar or antagonistic functions. For example, *MAVS* (mitochondrial antiviral signaling protein), a regulator of antiviral innate immunity, expresses two transcript isoforms, where the miniMAVS antagonizes the full-length MAVS to induce interferon production [32]. Here we calculated the frequency changes of ASEs with expression profiles of transcript isoforms. We observed that five (*CYP4F22, BTN, GSTA2, ADH5, and DHRS2*) genes showed significant differential ASEs in liver transcriptomes of overfed ducks compared with control ducks (Additional file 2: Fig S3).

**Fig. 5:**
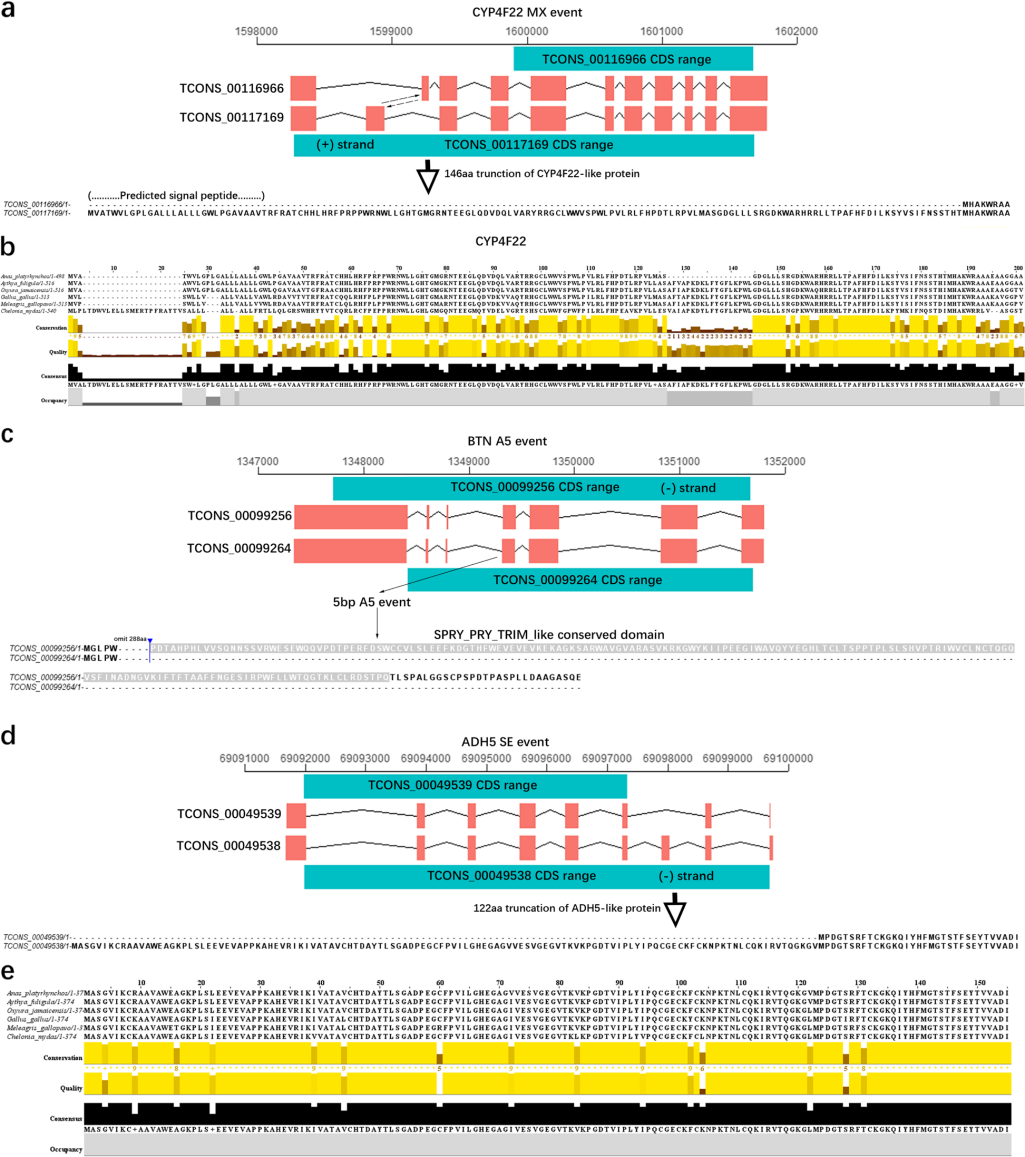
Analysis of transcripts related to lipid metabolism. **a** Structural comparison of a truncated transcript (TCONS_00116966) and full-length CDS (TCONS_00117169) for the *CYP4F22* gene in ducks (the top line is chromosome coordinates axis). **b** Multiple protein sequence alignment of CYP4F22 gene in ducks with five other birds. **c** Structural comparison of an A5 alternative transcript (TCONS_00099256) and full-length CDS for *BTN* gene in ducks **d** Structural comparison of a truncated transcript (TCONS_00049539) and full-length CDS for *ADH5* gene in ducks. **e** Multiple protein sequence alignment of *ADH5* gene in ducks with five other birds

*CYP4F22* (cytochrome P450 family 4 subfamily F member 22) as a fatty acid ω-hydroxylase involved in lipid metabolism to maintain the skin barrier in mice [33]. Signal peptides carry information for protein secretion and play an important role in human diseases [34–36]. Interestingly, we found overfed ducks preferred to express a transcript isoform with a signal peptide in its N-terminal, while control ducks preferred to express a transcript isoform without N-terminal signal peptide (Fig. 5a and Additional file 2: Fig S4a). We then evaluated the impact on biological function of an amino acid indel in transcript isoforms using PROVEAN software (score < 2.5 indicates a harmful detrimental change) [37]. This suggested that deletion of 146 amino acids at the N-terminal in *CYP4F22* transcript isoform TCONS_00116966 (with a score of -289.14), was suggested to be deleterious to *CYP4F22* function in ducks. We performed the cross-species alignment of CYP4F22 proteins in six birds and found the N-terminal 24 amino acids showed low conservation, while the remainder of the sequences were relatively conserved in the N-terminal 200 amino acids of CYP4F22 proteins (Fig. 5b and Additional file 3: Table S4). Since the transcript isoform of *CYP4F22* which missing the signal peptide are preferred in control ducks, we inferred that ASEs might regulate secretion or localization of CYP4F22 proteins by alternative splicing. We also noticed an A5 ASEs in a *BTN* gene, which had been reported to regulate milk-lipid secretion in mice [38]. The alternative transcript TCONS_00099256 encoded a 513aa (amino acid) longer protein and was expressed at lower levels, while TCONS_00099264 had a 209aa truncation of the cytoplasmic domain and was expressed more highly in overfed ducks when compared to that in control individuals (Fig. 5c and Additional file 2: Fig S4b). Furthermore, we found that the B30.2 domain was lost in the TCONS_00099264 encoding protein (Additional file 2: Fig S4c). *ADH5* (alcohol dehydrogenase 5 class-3, also called ADH-3) has been shown to protect the liver from the damage of nonalcoholic hepatic steatosis in mice [39]. We found an ASE event in the *ADH5* gene of ducks leading to a 122aa truncation in the N-terminal of the protein and having deleterious consequences on protein function (-459.358 Provean score) (Fig. 5d and Additional file 2: Fig S4d). Cross-species sequence alignment analysis showed ADH5 proteins were highly conserved (Fig. 5e). The frequency of this ASE event was lower in overfed ducks, thus overfed ducks had more full-length transcripts of ADH5 protein.

The *GSTA2* gene functions in detoxification of electrophilic compounds such as H_2_O_2_ or other products of oxidative stress [40]. The relative abundance of an A3 event in *GSTA2* was observed to be lower in control individuals (Additional file 2: Fig S3). However, no obvious change was identified during Phobius analysis of the alternative transcripts (TCONS_00036568) of the *GSTA2* gene (Additional file 2: Fig S4e and Additional file 2: Fig S5a). The results from Provean show limited expected harm of the short protein sequence change, suggesting a relatively slight effect of the protein truncation (-13.715 score). Therefore, this A3 event has less influence on *GSTA2* protein function. The *DHRS2* gene localizes in the mitochondria and plays a role in oxidation–reduction processes [41]. An N-terminal truncation by an ASE event was found in the *DHRS2* gene in ducks, with the frequency of this ASE event being lower in overfed ducks. Thus overfed ducks express more full-length transcript of the *DHRS2* gene (Additional file 2: Fig S3). We showed the selective N-terminal truncation of predicted DHRS2 protein sequences (Additional file 2: Fig S4f). The alignment of DHRS2 protein in duck with another five species shows that the 1-100aa region has relatively higher conservation (Additional file 2: Fig S5c). Provean predicts the potentially harmful result of this truncation with a score of -312.288. Under the overfed condition, ducks reduced levels of the truncated protein and increased expression of the full-length protein of the *DHRS2* gene.

## Discussion

Ducks provide a good model for the study of fatty liver. Overfeeding of energy-rich food in ducks quickly induced non-pathogenic fatty liver. We performed full-length transcript sequencing of sibling ducks to acquire full-length transcript isoforms and to further detect ASEs. We identified 77,237 transcripts in liver from overfed ducks and 69,618 transcripts in control ducks. The expressional profile and structural information of full-length transcripts were used to evaluate the relative abundance of ASEs in duck livers under different feeding conditions. The enrichment of ASEs in lipid metabolism related genes indicates transcript-level changes under the overfeeding condition.

Premature mRNA may produce different mature mRNA by ASEs. Our study provides us a group of differential ASEs between overfed and control ducks. ASEs with significantly differential abundance may reveal the regulation pattern of AS splicing involved in lipid metabolism. Signal peptides (16-30aa) are important in multiple fields such as protein secretion mechanisms and disease diagnosis [42]. *CYP4F22* plays an important role in producing acylceramide, which is a key lipid of skin barrier in mice [43]. The loss of signal peptide of CYP4F22 protein indicates that ASEs may cause alternative protein localization to influence lipid metabolism of ducks. The *BTN* (Butyrophilin) gene family was identified in lactating mammary gland and associated with lipid secretion [38, 44]. B30.2 is a classical conserved domain of *BTN* genes, possessing multiple functions including resisting virus invasion, regulating T cell activity, and lipid secretion [45–47]. The identified transcript isoform (TCONS_00099264) of the *BTN* family gene have lost the B30.2 domain in our study. The presence or absence of the B30.2 domain in the identified BTN protein may change the binding ability of the BTN protein. The structural changes in BTN protein products suggest that the lack of conservation of this domain or functional region is also a mode of regulation of lipid metabolism in duck liver.

Ducks may have unique mechanism to protect their liver from damage after lipid deposition and ASEs may play a key role in the protection process. Previous studies showed that oxidative stress induced by lipid accumulation was considered as one of the key factors for the exacerbation of NALFD [48, 49]. Glutathione (GSH) is a classical antioxidant substance, which can improve antioxidant defense ability. Increasing the level of glutathione is considered as one of the methods to treat NAFLD. Mice given glycine-based treatment recover from NAFLD, with glutathione accumulating in the process of treatment, indicating that glutathione can protect liver from NAFLD [50]. The ratio of GSH/GSSG (glutathione/oxidized glutathione) is a good marker for oxidative status of cells and high level of GSSG indicates the severe steatosis and oxidative stress in liver [51]. Studies have shown that the synthetic substrates (glycine and serine) of GSH were lower, and GSH level was decreased in NAFLD patients [52]. The concentration of GSSG in human was significantly increased and the GSH/GSSG ratio was lower with NAFLD [53]. The depletion of GSH means serious oxidative stress and probable injury in human liver. However, in waterfowl such as mule ducks, GSH is not depleted during the fatty liver period and the GSH/GSSG ratio is relatively higher compared with human [54]. The different dynamics of GSH compared with human might contribute to the non-pathogenic result of fatty liver in ducks, which was different from that of human NAFLD. Among genes detected with ASEs in duck, ADH5 protect glutathione from consumption of endogenous formaldehyde [55]. We found differential ASEs in the ADH5 gene of ducks, implying that this might regulate the ADH5 protein to resist the damage of oxidation. We also found alternative splicing in GSTA2 (glutathione S-transferase alpha 2) in ducks. GSTA2 functions in oxidative stress and protects cells from oxidation through combination with GSH [56]. These results suggested that alternative splicing may enhance antioxidant ability to avoid damage form fatty liver in ducks.

Our studies on ASEs shed further light on regulation of lipid metabolism and GSH metabolism at the transcript level and provide us with evidence of the potential factors leading to differential fatty liver disease processes in humans.

## Conclusions

Our study provides the full-length liver transcriptome of Pekin ducks to allow analysis of transcript structure. A total of 126,277 transcripts were generated and 27,317 ASEs identified, enabling us to further explore the events related to non-pathogenic fatty liver. ASEs of numerous genes involved in lipid metabolism were significantly changed by in ducks with fatty liver. Identified candidate genes *GSTA2*, *ADH5* and *DHRS2* are involved in oxidation resistance and ASEs might change their protein product to function in fatty liver process. The future challenge will be the functional validation of each transcript isoform involved in fatty liver in poultry and cross species experiments in mice. Taken together, our full-length transcriptome sequencing of overfed and control ducks enlightens us to the role of ASEs in the formation of and defense against fatty liver.

## Methods

### Animal feeding

Five sib-pairs of 11 week-old male ducks were reared at the Jiangsu Institute of Poultry Science, China and divided into two groups. The control group were fed with 180 g/d (gram/day) commercial feed to 14 weeks old. The overfed group were fed with 150 g corn twice a day on the first three days of the 12^th^ week and increasing to 200 g twice a day on the last four days of the 12^th^ week to adapt to the overfeeding condition. After the preparation of overfeeding at the 12^th^ week, the overfed group was fed 150 g corn three time a day until 14 weeks old. After 14 weeks feeding, the sib-pair ducks were euthanized by electronarcosis and cervical dislocation and then liver tissues were collected.

### PacBio full-length transcriptome library preparation and sequencing

Library construction was performed according to the PacBio official protocol of Huada Gene Co. Ltd. BGI (Beijing, China). Total RNA was extracted from liver tissue of a sib-pair ducks from control and overfeeding groups using Trizol reagent (ThermoFisher Scientific). After quality testing, RNA was reverse transcribed into cDNA by SMARTer™ PCR cDNA Synthesis Kit (Clontech, CA, USA). Full-length transcriptomic libraries were constructed to capture complete structure information. SMART primers were incorporated and PCR was performed for single stranded cDNA and double stranded cDNA in turn. Bluepippin (Sage Science, MA, USA) was used for cDNA library length classification and PCR amplification was performed again in a different cDNA library. Sequencing adaptors were linked to cDNA, and linear DNA without adaptor was removed. Finally, after quality tests using an Agilent 2100 (Agilent, CA, USA) and Qubit HS (Invitrogen, CA, USA), sequencing was carried out on the PacBio sequel platform (PacBio, CA, USA).

### Sequence processing

Full-length transcriptome raw sequencing data was strictly processed in accordance with the PacBio official smrtlink_5.1.0 work flow (https://www.pacb.com/support/ software-downloads). With this pipeline, CCS (Circular consensus sequencing) reads were generated and classified into full-length non-chimeric (FLNC) and non-full-length reads. FLNC reads were then passed through ICE (Iterative Clustering for Error Correction) and input into ICE Partial and Quiver, together with non-full-length reads to acquire unpolished reads. Reads were then polished using RNA-seq data with LoRDEC software (version 0.6) with parameters -k 19, -s 3 [57]. The polished reads were mapped to our recently developed high quality reference genome, SKLA1.0 (NCBI BioProject accession number PRJNA792297) by minimap2 with parameter -ax splice -uf [58]. The redundant results of minimap2 were removed by cupcake (version 28.0.0) with parameter -c 0.85 -i 0.9 –dun-merge-5-shorter (https://github.com/Magdoll/cDNA_Cupcake). Transcripts from ducks under different feeding conditions were merged non-redundantly for subsequent analysis. Sqanti3 was used to evaluate and annotate the long-read transcriptome [59]. Sqanti3 transcript evaluation was performed using default parameters. Associated reference genes of each transcript and different types of splice junction were identified and classified by Sqanti3. Fusion transcript were also identified by Sqanti3 and the distance between transcript members in one fusion must be greater than 10,000 bp.

### LncRNA prediction

CNCI (Coding-Non-Coding Index), CPC (Coding Potential Calculator) and GeneMark were used for transcript coding potential identification [60–62]. The non-coding transcripts identified by all three algorithms were filtered using thresholds of ORF < 100aa and transcript length > 200nt (nucleotide). ORF sequences were acquired from transdecoder (ver 5.5.0) (https://github.com/TransDecoder/Trans Decoder). Pfam domain and super family prediction was implemented and transcripts found by Pfam database were eliminated. A region 10,000 bp upstream and downstream of lncRNA in the DET set was regarded as the maximum cis-acting screening window, and coding genes within this range were inferred as cis-acting target genes. Pearson correlation was performed to test reliability of cis-acting pairs and the pairs within one gene range were excluded.

### Differential transcript analysis

The transcript-level expression was calculated by the Kallisto software (version 0.48.0) with default parameters based on short reads and full-length transcript sequences [63]. Kallisto uses pseudoalignment framework and can quantify the expression of transcripts without additional alignment or reference genome. Transcript expression level in TPM (transcript per million) was used for significantly differentially expressed transcript screening through the sleuth R package with a threshold of *p*-value < 0.01 [64]. Differentially expressed transcripts were annotated by eggNOG webtools and GO enrichment analysis was performed by DAVID with FDR < 0.05 [65, 66]. KEGG enrichment analysis was performed by KOBAS online tools with *p*-value < 0.01 [67]. The data used for KEGG enrichment originates from KEGG pathway database (https://www.kegg.jp/kegg/pathway.html) [68].

### Detection and analysis of alternative splicing events

Alternative splicing (AS) event analysis was implemented by suppa2 software (version 2.3) with parameters: -e SE SS MX RI -f ioe [69]. The combination of identified ASEs and transcript-level expression were used to screen out significant ASEs by suppa2 with *p*-value < 0.05. The Phobius software (https://phobius.sbc.su.se/) was used to predict transmembrane topology and signal peptides. The NCBI CDD tool was used to predict conserved domains. The protein sequences of all transcripts of fatty acid related genes with ASEs were acquired from ORFfinder and the redundant protein sequences were removed. The predicted deleteriousness of protein sequences changes was evaluated by Provean [37]. Protein sequences were downloaded from the NCBI website and multiple sequence alignment was performed using the Prank tool (version 170,703).

## Supplementary Information

**Additional file 1.**

**Additional file 2.**

**Additional file 3.**

## Data Availability

The original data files have been uploaded to the NCBI SRA database. The full-length transcriptome of livers of overfed and control ducks can be accessed under accessions SRR20724681 and SRR20724682. The accession numbers for the RNA-seq data are SRR20707313-SRR20707319, SRR20707330, SRR20707341, SRR20707342. Moreover, we also generate a reviewer link (https://dataview.ncbi.nlm.nih.gov/object/PRJNA863477?reviewer=4cr4hpj0egsuihvqqqeo6ctnnc). The genome draft had been assigned the following accession number JAKEIL000000000 by NCBI website (PRJNA792297, https://dataview.ncbi.nlm.nih.gov/object/PRJNA792297?reviewer=us7cqb9blqt4v5po2d8rdpj77h).

## Abbreviations

AS: Alternative splicing
lncRNA: Long noncoding RNA
DET: Differential expressed transcripts
GO: Gene ontology
KEGG: Kyoto Encyclopedia of Genes and Genomes
RNA-Seq: RNA sequencing
ORF: Open reading frame
ASEs: Alternative splicing events
CYP4F22: Cytochrome P450 family 4 subfamily F member 22
FASN: Fatty acid synthase
FADS: Fatty Acid Desaturase 1
GSTA2: Glutathione S-transferase alpha 2
ADH5: Alcohol dehydrogenase 5 class-3
DHRS2: Ehydrogenase/reductase 2
BTN: Butyrophilin
XOR: Xanthine dehydrogenase
ENPP1: Ectonucleotide pyrophosphatase 1
SERPINA1: Serpin family A member 1
MGAT2: Alpha-1,6-mannosyl-glycoprotein 2-beta-N-acetylglucosaminyltransferase
SSU72: RNA polymerase II CTD phosphatase
