# Supplementary material for: Identification of alternative splicing events related to fatty liver formation in duck using full-length transcripts
Source: BMC Genomics. 2023 Mar 1;24:92. doi: 10.1186/s12864-023-09160-4 (PMC9976415; doi:10.1186/s12864-023-09160-4)
Supplement: Supplementary file 2 — Additional file 2. [file 12864_2023_9160_MOESM2_ESM.pdf]

Supplementary Figures

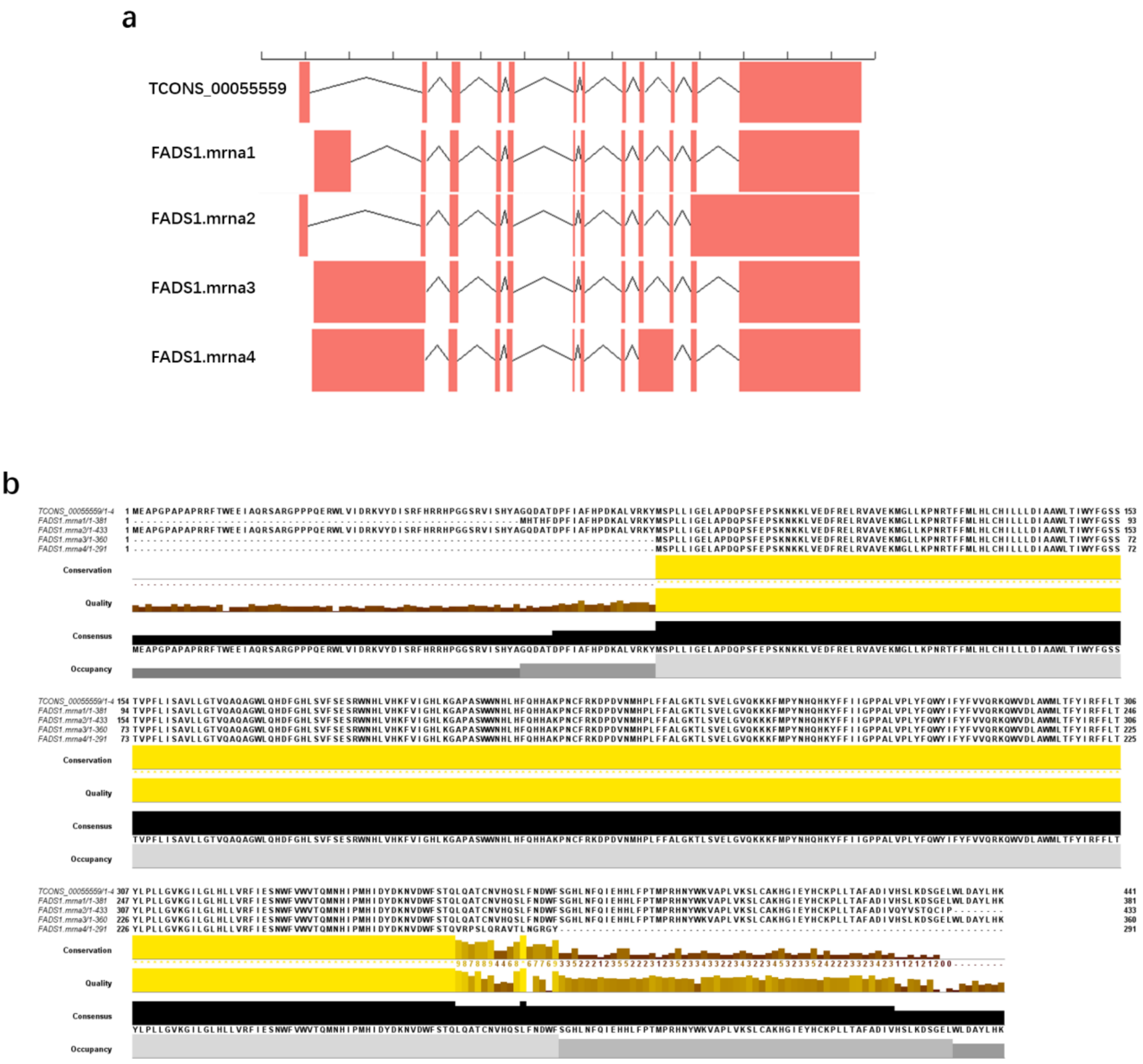

Fig. S1 Structural comparison and sequence alignment of FADS1 novel transcript TCONS_0005559 and known transcripts in duck. a. Exon distribution of reference FASN transcripts and TCONS_0005559. b. Protein sequence alignment of FASN transcripts and TCONS_0005559.

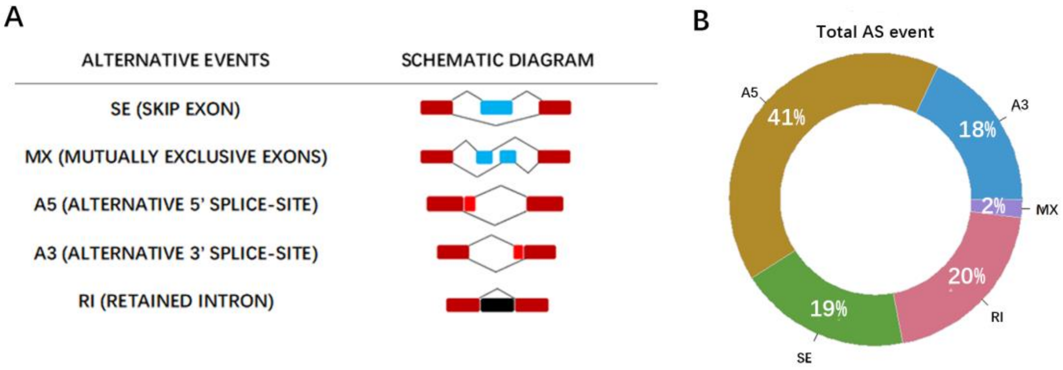

Fig. S2 Classes and ratio of ASEs detected in this study. a. ASE classes detected in this study. b. Ratio of each class of ASEs.

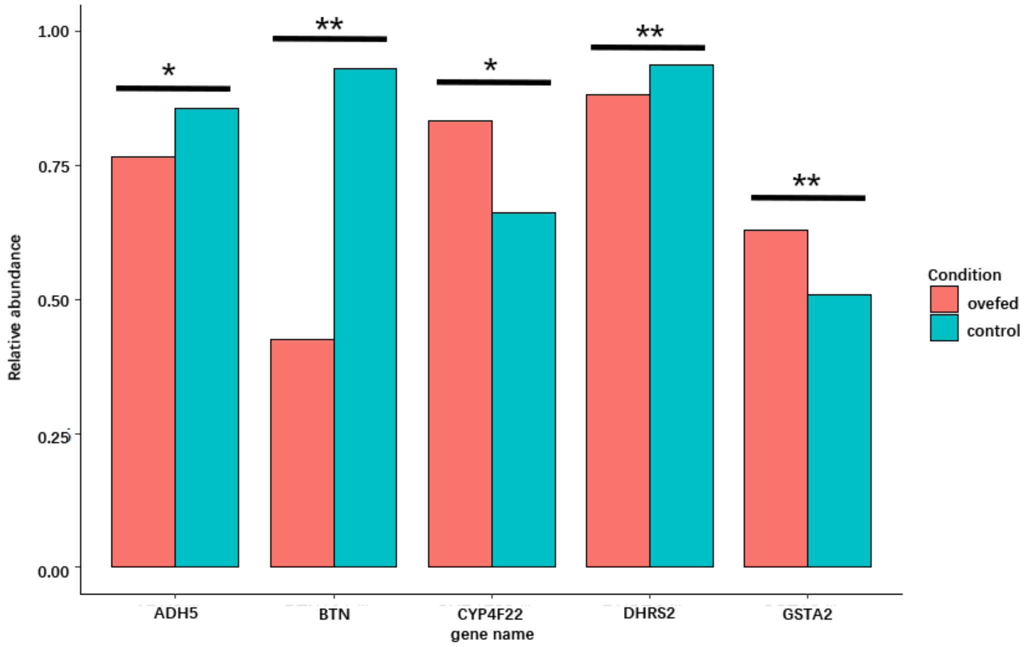

Fig. S3 Relative abundance of each ASE in five genes.

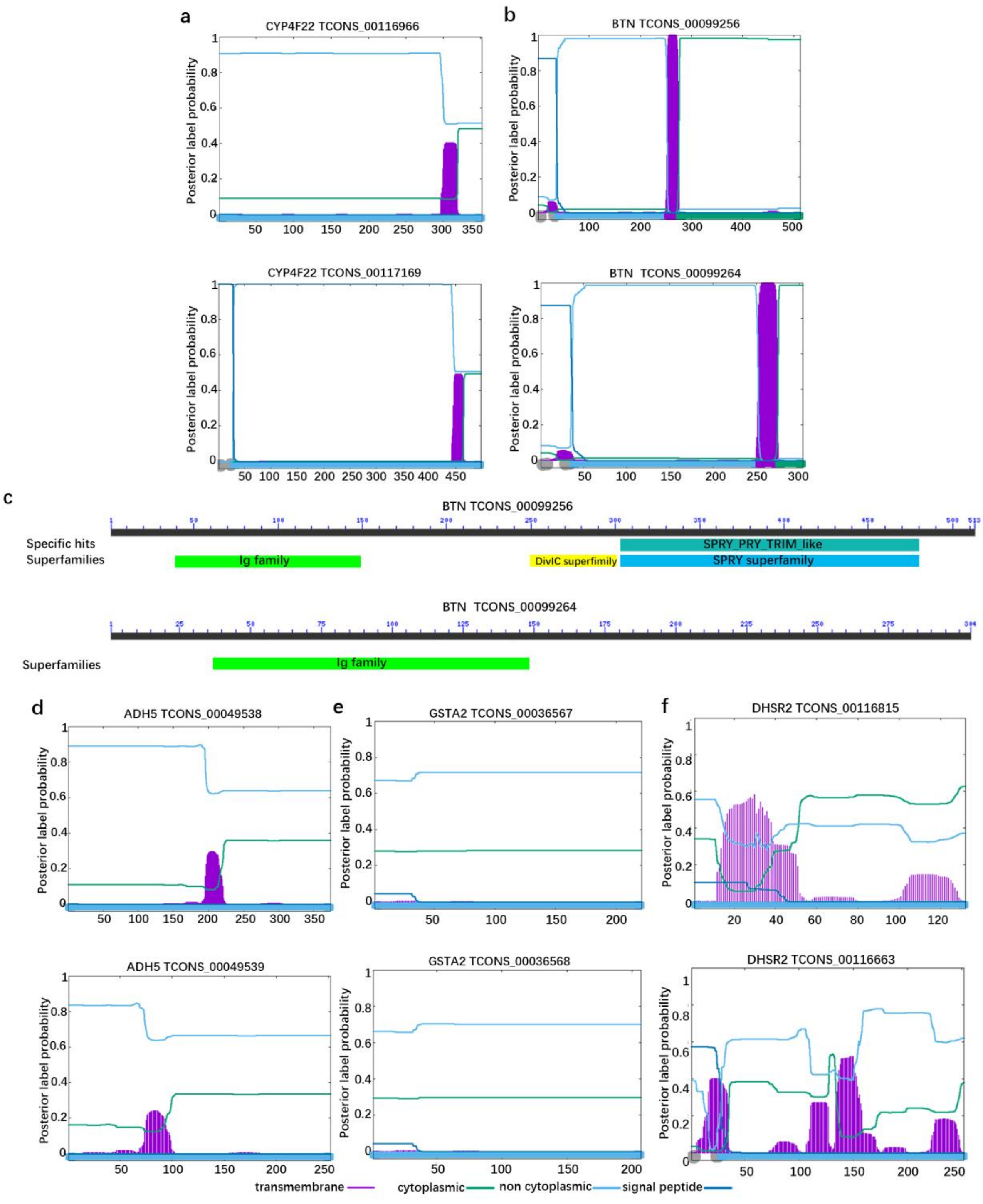

Fig. S4 Prediction of Phobius transmembrane topology, signal peptide and CDD tools motif of transcripts from five genes. a-b. Transmembrane topology and signal peptide of CYP4F22 and BTN alternative transcript and full-length CDS. c. Motif of BTN proteins. d-f. Transmembrane topology and signal peptide of ADH5, GSTA2, DHRS2 alternative transcript and full-length CDS.

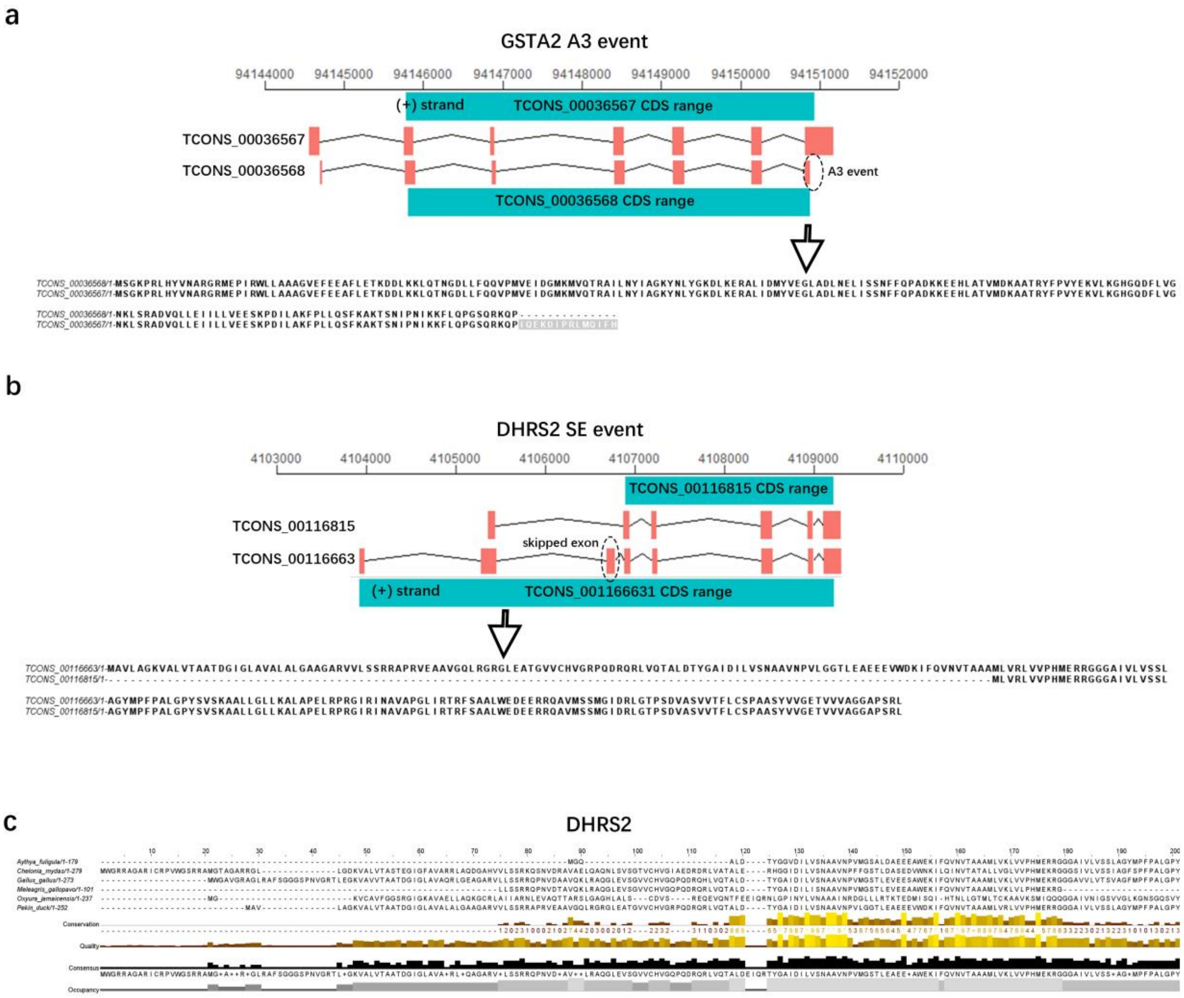

Fig. S5 ASE analysis of *GSTA2* and *DHRS2* genes. a. Sketch Map of ASEs in *GSTA2* genes and the influence on CDS. b. Sketch Map of ASEs in *DHRS2* genes and the influence on CDS. c. Protein sequence alignment of DHRS2 transcript TCONS_00116663 and full-length CDSs from five other birds.
